# Effects of bovine spermatozoa preparation on embryonic development in vitro

**DOI:** 10.1186/1477-7827-4-58

**Published:** 2006-11-13

**Authors:** Marko Samardzija, Martina Karadjole, Iva Getz, Zdenko Makek, Marijan Cergolj, Tomislav Dobranic

**Affiliations:** 1 Clinic for Obstetrics and Reproduction, Faculty of Veterinary Medicine, University of Zagreb, Heinzelova 55, Zagreb, Croatia

## Abstract

The aim of our research was to examine the ability of density gradient preparation BoviPure^® ^and swim up method on bull sperm separation and in vitro embryo production (IVP) systems. Frozen/thawed semen from six Simmental bulls was pooled and treated using both methods. The sperm motility, concentration, membrane activity, membrane integrity and acrosomal status were evaluated and compared before and after sperm processing using BoviPure^® ^and swim up methods. We also evaluated and compared cleavage rates, embryo yield and quality between the methods. There were significant differences (P < 0.05) between the sperm characteristics before and after BoviPure^®^, but not after swim up method. However, there were significant differences for sperm results among those two mentioned methods. A total of 641 oocytes were matured and fertilized in vitro and cultured in SOFaaBSA. The percentage of cleavage (Day 2) and the percentage of hatched embryos (Day 9) were similar for both methods. However, embryo production rate (Day 7) was significantly higher using BoviPure^® ^method (P < 0.05). Also, total cell number and embryo differential staining (inner cell mass and trophectoderm cells) of Day 7 morulas and blastocysts showed that BoviPure^® ^treated sperm displayed higher quality embryos compared to swim up method (P < 0.05). Our results indicate that BoviPure^® ^method has an enhanced capacity in sperm selection for in vitro embryo production when compared with swim up method. So, we concluded that BoviPure^® ^could be considered as a better alternative to swim up method for separating bull spermatozoa from frozen/thawed semen for IVP of bovine embryos.

## Background

Mammal spermatozoa have very expressive heterogeny in morphology, motility and nuclear stability. During copulation, cervical mucus represents a barrier which allows only migration of progressively motile spermatozoa with normal morphology and high nuclear stability [1]. Frozen bull spermatozoa after thawing have lower percentage of progressive motility (30–70%), but percentage of morphologically normal spermatozoa in thawed ejaculate is equal to fresh semen [2]. Sperm separation procedures are able to significantly improve the sperm quality with higher rate of progressive motility and morphologically normal spermatozoa. In the in vitro production of embryos, sperm separation methods have very important role. Such selection of spermatozoa separates motile sperm from nonmotile, removes seminal plasma, cryoprotective and infectious agents, other background materials and debris [3,4] and also in the same time initiates the capacitation of sperm [5]. The morphological selection of spermatozoa in the prepared population varies, mostly with tail and midpiece defects being primarly excluded.

Many sperm separation methods have been developed to improve sperm quality based on high rate of progressive motility and morphologically normal spermatozoa. Some of the most important sperm separation methods are: selective fractionation of subpopulations (density-gradient centrifugation) and self-migration techniques swim-up [1]. The efficacy of sperm preparation methods could be evaluated using different sperm parameters such as sperm motility, morphology, concentration, viability, membrane activity, acrosomal status, reactive oxygen species (ROS) formation, chromatin maturity and integrity, protamination degree and IVP rates [6-9]. BoviPure^® ^is a commercial medium for the density-gradient centrifugation of bull spermatozoa. It is an iso-osmotic salt solution containing colloidal silica particles coated with silane specifically formulated for use with bull sperm. At this time, very few studies have been conducted to evaluate BoviPure^® ^for in vitro production of bovine embryos [9,10]. In contrast, swim up method is routinely used for many years in *in vitro *procedures of bovine embryos [6,11-13]. Comparing swim up method and Percoll gradient Parrish et al. [2] obtained similar sperm results for both methods, although a lower concentration resulted for swim up method. However, the fact that cleavage rate was significantly higher for swim up method compared to Percoll compensated a lower sperm concentration results. With swim up method we can safely separate spermatozoa based on their motility and morphology [1]. Research that compared these two methods (BoviPure and swim up) of bull sperm preparation for *in vitro *production of bovine embryos was not done until today. The present study was designed to compare the efficiency of two sperm separation methods evaluating sperm quality parameters and subsequent development and quality of bovine IVP embryos.

## Methods

### General approach

For the purpose of our research a group of six Simmental bulls with proven fertility was chosen. Frozen-thawed sperm of all the six bulls was pooled and then the sperm parameters were estimated. Chemicals were purchased from Sigma Chemical Co. (St. Louis, MO, USA) unless otherwise stated.

### BoviPure^® ^gradient

Sperm preparation for in vitro fertilization (IVF) on BoviPure^® ^gradient was accomplished according to producer's directions (Nidacon International AB, Göthenborg, Sweden). BoviPure^® ^works at room temperature. In a 10 mL centrifuge tube 2 mL of BoviPure^® ^Bottom Layer Medium was placed and then carefully layered with 2 mL of BoviPure^® ^Top Layer Medium. Aliquots of 400 μL of thawed semen were gently placed into a warm test tube and diluted with BoviPure^® ^Extender in 1:1 ratio. The amount of 800 μL of the prepared semen was gently loaded onto the top of the gradient and centrifugated for 20 min at 300 × *g*. After centrifugation, the fluid above the sperm pellet was carefully removed. The pellet was resuspended with 5 mL of BoviPure^® ^Wash and centrifugated for 10 min at 500 × *g*. This final pellet was resuspended in 150 μL of TALP (Tyrode's albumin lactate pyruvate medium), and the final sperm concentration was adjusted to 1 × 10^6^spz/mL.

### Swim-up method

Swim up was performed as described previously according to Shamsuddin et al. [11] with minor modifications. Briefly, 400 μL were placed under 1 mL TALP, and incubated at 39°C, 5% CO_2 _and maximum humidity. After 1 h, 1 mL of the upper fraction were collected and placed in 3 mL of TALP, centrifuged (200 × g, 10 min). The pellet was then resuspended with 3 mL TALP and centrifugated for 10 min at 200 × *g*. Afterwards the pellet was resuspended in 150 μl TALP and the final sperm concentration was adjusted to 1 × 10^6^spz/mL.

### Sperm quality parameters assessment

The sperm quality parameters were evaluated immediately after thawing and after sperm preparation for IVF. Sperm concentration was determined using a Thoma chamber. Progressive motility of semen was subjectively assessed by visual estimation under inverted microscope. The functional integrity of bovine sperm membrane was determined by hypoosmotic swelling test (HOS) and dual staining with SYBR-14/PI. The hypoosmotic swelling test was performed according to Jeyendran et al. [14] with the exception that osmolarity was adjusted to 100 mOsm/kg as described for frozen-thawed bovine spermatozoa by Correa and Zavos [15]. The assay was performed by mixing 50 μL of semen with 1 mL of hypoosmotic solution and incubating at 37°C for 60 min. A total of 400 cells were evaluated in at least five different fields under 400 × magnification. Spermatozoa with changes were denoted as swelled or HOS positive (HOS+). To assay the sperm viability we used a SYBR-14/PI staining as described by Januskauskas et al. [16]. Aliquots of 50 μL thawed semen were diluted in 150 μL of mTALP containing 3 μL PI and 2 μL SYBR-14 (Live/Dead Sperm Viability Kit, Molecular Probes Inc., USA). Incubation and staining procedures of the samples were performed according to the method described by Garner and Johnson [17] with minor modifications. The nuclei of SYBR-14-stained live spermatozoa were bright green, while dead sperm nuclei were stained red with PI (propidium iodide). A total of 300 spermatozoa were counted under 400 × magnification in two replicates, and the mean values were then used for the analysis. For acrosome staining, a slightly modified procedure described by Januskauskas et al. [16] was used. Aliquots (15 μL) of ethidium homodimer (EthD-1) counter-stained semen were smeared onto microscope slides, air dried, fixed and permeabilized with 96% ethanol for 30 s. The unbounded dye of EthD-1 was removed using centrifugation at 200 × *g *for 5 min twice, preventing that excess of dye stain live spermatozoa after permeabilization with ethanol. We kept smear for 15 min at -20°C and then eliminated the ethanol. Twenty microliters of FITC-labeled pisum sativum agglutinin (FITC-PSA) solution (100 μg/mL) in PBS were spread over each smear and incubated in moist chamber at 37°C for 7 min. Smeared slides were agitated in destilled water to remove unbound dye, air dried and mounted with 15 μL of anti-fade solution. Three hundred morphologically normal spermatozoa were assessed under 1000 × magnification in each smear and then classified according to the method of Sukardi et al. [18] in one of four categories, based on their FITC-PSA and EthD-1 staining patterns: (a) live, acrosome intact sperm; (b) dead, acrosome intact sperm; (c) live, acrosome reacted sperm; (d) dead, acrosome reacted sperm.

### Collection of cumulus-oocyte complexes (COC) and in vitro maturation (IVM)

Bovine ovaries were collected at local abattoir and transported to the laboratory in physiological saline (0.9%) with antibiotics (100 I.U. penicillin and 100 μg streptomycin/mL) at 37°C within 3 h after slaughtering. Cumulus-oocyte complexes (COCs) were aspirated from 2 to 8 mm diameter follicles using 18G needles attached to a vacuum pump. Only oocytes with homogenous ooplasm and intact cumulus investment were selected for further development procedure. The oocytes were washed three times in TCM 199 medium buffered with 15 mM HEPES supplemented with 10% of FCS and then three times in IVM medium. In vitro maturation medium consisted of TCM 199 bicarbonate medium supplemented with 10% FCS, FSH/LH (Pergonal^® ^75/75 I.U./mL, Serono), 1 μg/mL estradiol-17β and 100 μM cysteamine. Oocytes were incubated in groups of 10 in 50 μL droplets of maturation media under mineral oil at 39°C with 5% CO_2 _in air for 24 h.

### In vitro fertilization and culture (IVF and IVC)

The expanded COCs were washed in TALP-HEPES medium supplemented with 3 μg/mL BSA-FAF and transferred in 40 μL droplets of IVF medium under mineral oil. The COCs (n = 641) were randomly distributed in two groups (323 for BoviPure^® ^and 318 for swim up). Both sperm preparation methods were used on each day of IVF. The IVF medium was modified Tyrode's bicarbonate buffered solution supplemented with 10 μg/mL heparin, 0.5 μg/mL hypotaurine, 0.5 μg/mL epinephrine and 6 mg/mL BSA. The sperm suspension was then added at a volume of 10 μL to the droplets with oocytes. Sperm with COCs were co-incubated at 39°C in an atmosphere of 5% CO_2 _in air for 18–24 h. Fertilized oocytes were denuded by repeated pipetting from cumulus cells and spermatozoa and then washed three times in HEPES-TALP medium and in culture medium. Synthetic oviductal fluid (SOF) with amino acids and 8 mg/mL BSA, according to Edwards et al. [19] was used. Fertilized oocytes were cultured in vitro in SOF medium without glucose for 48 h and then transferred in SOF with 1.5 mM glucose and cultured in vitro until Day 9 at 39°C in 5% CO_2_, 7% O_2 _in 88% N_2_, according to Furnus et al. [20]. The medium was changed every 48 h. Bovine embryos were evaluated according to the IETS standards: on the 2nd day of culture we registered the number of cleaved embryos, on the 7th day the number of morulas and blastocysts and on the 9th day the number of hatched blastocysts [21].

### Differential staining of blastocysts

A random samples of Day 7 expanded blastocysts from both sperm separation protocols (12 for BoviPure^® ^and 12 for swim up) were double stained. The zona of blastocysts were removed by treatment with 0.5% pronase. Zona-free embryos were washed five times in PBS containing 0.1% PVA. Embryos were then incubated in a 30:70 dilution of rabbit anti-bovine whole serum in TCM 199 bicarbonate at 39° for 1 h. After washing in PBS 0.1% PVA, the embryos were incubated in a 1:4 dilution of a guinea pig complement in TCM 199 bicarbonate supplemented with 10 μg/mL propidium iodide (PI) for 1 h. The embryos were then briefly washed in ice-cold TCM 199 Hepes supplemented with 10 μg/mL PI and fixed into ice-cold absolute ethanol. After fixation, the embryos were transferred for 3–5 minutes to 10 μg/mL bisbenzimide in absolute alcohol at room temperature. Presumptive stained blastocysts were transferred to a drop of glycerol on a microscopic slide and covered with a cover slip. Embryos were examined under a fluorescence microscope (Olympus, Tokyo, Japan) equipped with UV filter. Bisbenzimide-stained inner cell mass (ICM) nuclei labeled with bisbenimide appeared blue and trophectoderm (TE) nuclei labeled with both bisbenzimide and PI appeared red or pink. The ICM and TE nuclei were counted under the microscope.

### Statistical analyses

The statistical analyses between methods were done by ANOVA (StatSoft, Statistica, 7.1 version 2005) using the arcsin transformation (arcsin√ *P*/100) of the percent values, comparisons by the Tukey's tests post hoc analysis and correlation analyses between sperm parameters before and after processing and total embryo yield (cleavage, morulas and blastocysts and hatching rate), were recorded at Day 2, 7 and 9, respectively.

## Results

**Figure 1:**
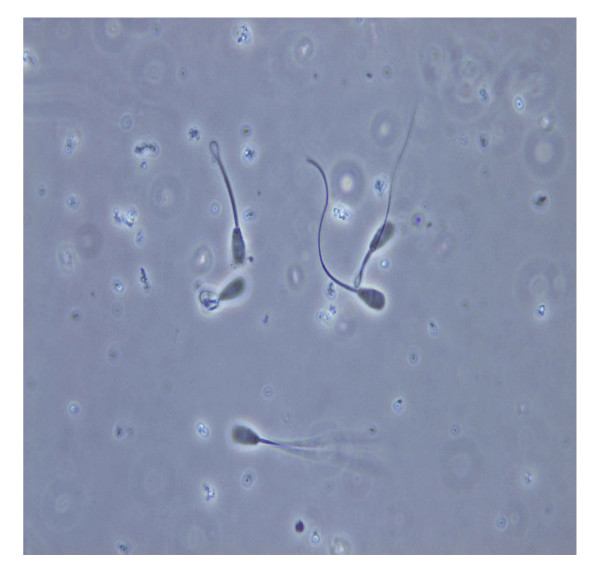
Inactive sperms and different swelling patterns of the active sperms after HOS test (1000×).

**Figure 2:**
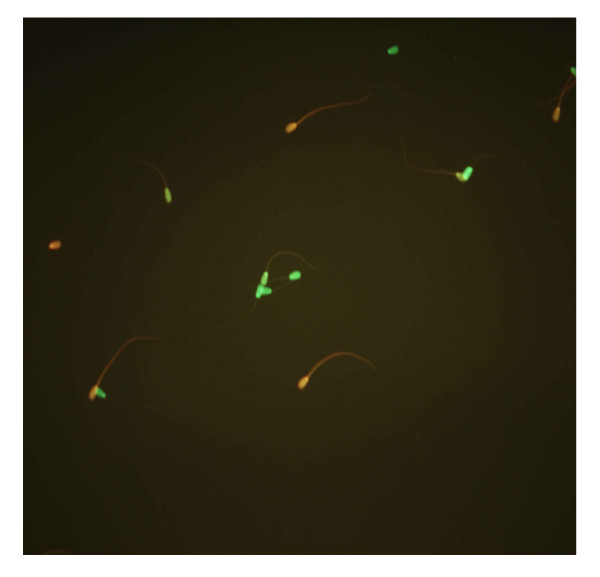
Live (green) and dead (red) sperms after SYBR-14/PI test (400×).

**Figure 3:**
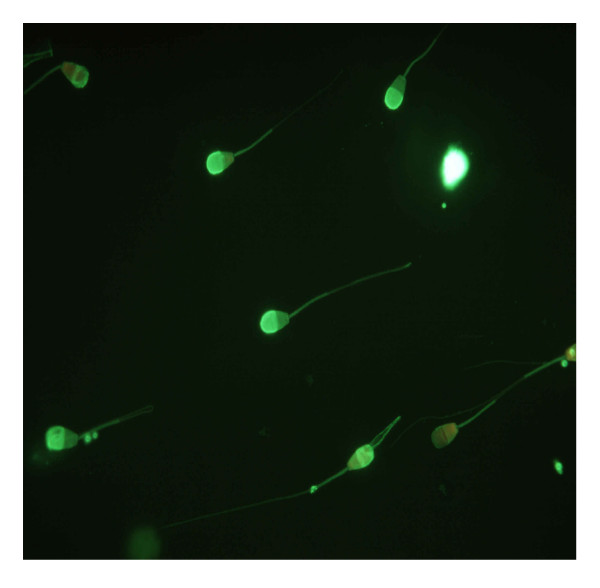
Live with intact acrosoma and dead spermatozoa without acrosoma after EthD/FITC-PSA test (1000×).

**Figure 4:**
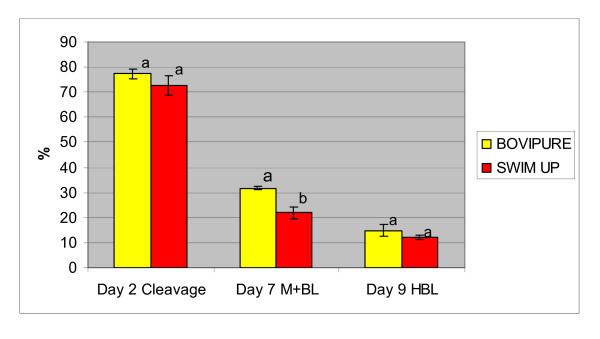
Cleavage, blastocysts and hatched blastocysts rates for BoviPure^® ^and swim up (mean ± S.E.M.), Values with different superscripts within the same columns differ significantly (P < 0.05)

The results of sperm parameters were shown in Table 1. Comparing the results of sperm motility before processing with the results after sperm processing it was found that there were significant differences (P < 0.05) between initial sperm and sperm after BoviPure^® ^method. Also, there were significant differences (P < 0.05) in the motility values between the sperm preparation methods. Comparing the results of sperm concentration before processing with the results after sperm processing it was found that there were significant differences (P < 0.05) between them. However, there were no significant differences for the concentration values between the sperm preparation methods. The significant differences were found (P < 0.05) between the sperm evaluation parameters before and after the processing with BoviPure^® ^method for HOS (Fig. 1), SYBR-14/PI (Fig. 2) and EthD-1/FITC-PSA (Fig. 3) tests. Also, there were significant differences (P < 0.05) in the mentioned tests between the sperm preparation methods. The IVF and IVC results are shown in the Fig. 4. A total of 641 oocytes (323 for BoviPure^® ^and 318 for swim up) were matured and fertilized in vitro and cultured in SOFaaBSA in six replications. The oocytes cleavage rate was 77.25 ± 2.02% for BoviPure^® ^and 72.63 ± 3.98% for swim up. The percentage of morulas and blastocysts on the 7th day of the in vitro culture were 31.79 ± 0.71% for BoviPure^® ^and 21.91 ± 2.49% for swim up. The results of hatched blastocysts on the 9th day of the culture were 14.88 ± 2.38% for BoviPure^® ^and 12.11 ± 0.69% for swim up. The IVF and IVC results were compared and no significant differences between the methods in cleavage (Day 2) and hatched blastocysts (Day 9) rates were revealed. However, the number of morulas and blastocysts (Day 7) did differ significantly between sperm separation methods (P < 0.05). Total cell number and embryo differential staining (inner cell mass and trophectoderm cells) of Day 7 blastocysts showed that BoviPure^® ^treated sperm displayed better quality embryos compared to swim up method (P < 0.05). Effect of BoviPure^® ^and swim up methods on total cell number and number of inner cell mass cells in Day 7 blastocysts are shown in Table 2.

**Table 1:** Sperm parameters results (means ± S.E.M.)

Sperm separation protocol	Progressive motility (%)	Concentration (10^6^Ml)	HOS % active	SYBR-14/PI % live	EthD-1/FITC-PSA % live with intact acrosome
Initial (n = 6)	50.00 ± 8.16^a^	82.75 ± 5.25^a^	39.94 ± 8.98^a^	43.34 ± 6.88^a^	46.04 ± 12.56^a^
BoviPure (n = 6)	70.00 ± 3.54^b^	27.25 ± 1.70^b^	54.35 ± 2.75^b^	72.68 ± 2.79^b^	75.93 ± 0.91^b^
Swim up (n = 6)	53.75 ± 3.15^a^	20.00 ± 5.34^b^	45.90 ± 1.84^a^	50.99 ± 2.18^a^	59.24 ± 2.42^a^

Values with different superscripts within the same columns differ significantly (P < 0.05)

**Table 2:** Effect of BoviPure^® ^and swim up methods on total cell number and number of inner cell mass (ICM) cells in day 7 blastocysts (mean ± S.E.M.)

Sperm	Total cells	ICM	
preparation methods	n	n	Proportion (%)

BoviPure^® ^(n = 12)	141.83 ± 6.14^a^	39.92 ± 2.83^a^	28.15^a^
Swim up (n = 12)	121.92 ± 7.32^b^	31.83 ± 2.13^b^	26.11^a^

Values with different superscripts within the same columns differ significantly (P < 0.05)

## Discussion

Considerable research evaluating the effects of bovine sperm preparation methods such as swim up and density gradient centrifugation have been done. Most of them referred to Percoll^® ^gradient [22-24]. The problem is that some batches of Percoll^® ^have endotoxic effect so it was discarded for use in assisted reproduction technics in human medicine [25]. There have been reports that batches of Percoll^® ^differ in composition and this variation may affect cleavage rates and embryo development [26]. As a result of Percoll^® ^endotoxicity many pharmaceutical companies researched for a good quality substitute for Percoll^® ^like Bovipure^®^. Bovipure^® ^is a sperm separation and purification product formulated specifically for use with bull sperm. Our results indicate that BoviPure^® ^was more effective at preparing sperm for IVF when compared to swim up (P < 0.05) regarding sperm motility 70.00 % vs. 53.75 %, respectively. Our results of sperm motility for swim up method are congruent to Risopatron et al. [27]. Mentioned authors compared two sperm separation methods and gained significantly higher percentage of motility for swim up (54.00%) compared to washing method (43.50%). Sperm viability, membrane activity and acrosome status evaluated by HOS, SYBR-14/PI and EthD-1/FITC-PSA tests showed a higher percentage of live and acrosome intact spermatozoa obtained after BoviPure^® ^method compared to swim up. Somfai et al. [24] used the dual stain to evaluate the viability and acrosome integrity of frozen-thawed bull sperm before and after Percoll^® ^and swim up methods. They found significantly increased proportion of live spermatozoa with intact acrosomes for Percoll^® ^(88.20%), than after the swim up method (69.40%) which is similar to our results obtained after comparing density gradient BoviPure^® ^and swim up method. Piomboni et al. [28] found that swim up selection based on sperm motility excludes many sperm with reacted acrosome and broken plasma membrane which was not established in our research. Determining sperm viability for swim up method Risopatron et al. [27] using dual staining found 63.20% of live sperm with intact membrane, while using washing method they found 53.20% of live sperm with intact membrane. Samardzija et al. [9] used HOS, SYBR-14/PI and EthD-1/FITC-PSA tests for determination of sperm viability, membrane activity and acrosome status for BoviPure^® ^and Percoll^® ^gradients. They found no differences between Percoll^® ^and BoviPure^® ^methods in percentage of live spermatozoa with intact acrosomes. It should be mentioned that the force of centrifugation might affect sperm motility and membrane integrity in bulls [29] and also in rams [30]. Because of that the sperm parameters results in those two protocols should be interpreted with caution. In the present research we compared the cleavage rates, embryo yields and quality for the both sperm separation methods and found no significant differences between methods in cleavage and hatched blastocysts rates. However, the number of morulas and blastocysts on Day 7 of culture was significantly higher for BoviPure^® ^method. We did not find high, significant correlations between the sperm parameters and embryo yield, except for acrosome status after EthD-1/FITC-PSA test (r>0.7;P < 0.05). That could be explained with the fact that spermatozoa with intact acrosomes were not capacitated. Non-capacitated spermatozoa showed a significant, although low relation to fertility [31,32]. Sieren and Youngs [10] used BoviPure^® ^method and obtained 77.20% of the cleaved oocytes and 21.60% of the blastocysts. The above mentioned authors evaluated the effect of coincubation of oocytes with frozen/thawed bull sperm using the BoviPure^® ^method and compared the same effect with a modified Brackett-Oliphant medium as a control group in the in vitro production of bovine embryos. The preparation of the bull sperm by the BoviPure^® ^method did not show significantly better effects on the cleavage rate (77.20%) and on the percentage of blastocysts on Day 8 of in vitro culture (21.60%) in comparison with the control group (71.90 and 17.10%). Those results demonstrated that the preparation of the bull sperm by BoviPure^® ^method did not significantly improve the ability of obtaining the bovine embryos in procedures in vitro. That is not consistent to our results because we established that bovine embryo development appeared to be superior following BoviPure^® ^compared to swim up method. However, we did not observe differences in cleavage rates and percentage of hatched blastocysts which was similar to results of mentioned authors. Samardzija et al. [9] examined the effect of BoviPure^® ^and Percoll^® ^on bull sperm separation for IVP of bovine embryos. They found no significant differences regarding sperm evaluation parameters between the methods. The cleavage (Day 2) and blastocysts (Day 7) rates were significantly higher (P < 0.05) for the BoviPure^® ^group compared to the Percoll^® ^group: 75.80 and 28.21%; 61.58 and 20.83%, respectively. However, the number of hatched blastocysts (Day 9) did not differ significantly between sperm separation methods. Our previous work indicates that BoviPure^® ^is acceptable method for sperm separation in bovine IVP which was in accordance to our results. In our research significantly higher blastocyst rates in BoviPure^® ^group vs swim up group make us suggest that BoviPure^® ^method allowed a faster cleavage and blastocyst yield than swim up method. Other explanation of different embryo yield could be that density gradient method selects spermatozoa with more compacted chromatin and less nuclear DNA damage than swim up method [33]. Embryos from BoviPure^® ^treated group displayed significantly higher total cell number comparing to swim up group. Cesari et al. [34] compared two bull sperm separation methods and revealed a significantly higher number of inner cell mass cells in Percoll treated group compared to swim up. Similar results were found by Rho et al. [35] who reported that goat blastocysts obtained from the Percoll treatment group had significantly more cells compared to swim up group (167 ± 5 vs 149 ± 4, respectively). Our study also demonstrated that BoviPure^® ^method resulted in significantly higher number of inner cell mass cells compared to swim up method which can be correlated with embryo quality. Although differences were found in cell counting, sperm treatment did not affect hatching rates. Our research showed predominance of sperm preparation by BoviPure^® ^in terms of blastocyst formation, total cell number and allocation of ICM. Lane and Gardner [36] reported that mouse fetal development after transfer was positively correlated with number of blastocyst cells and with ICM development, but not with number of TE cells or hatching ability. Therefore, it would be advisable to extend the comparisons of these two sperm preparation methods by embryo transfer into recipient cows which will allow for more reliable resultes of subsequent embryo development.

## Conclusion

Our results indicate that BoviPure^® ^method has an enhanced capacity of selected sperm for embryo production when compared with swim up method. So, we concluded that BoviPure^® ^could be considered as a better alternative to swim up method for separating bull spermatozoa from frozen/thawed semen for in vitro production of bovine embryos.
